# Atraumatic Splenic Rupture as an Unusual Presentation of Hairy Cell Leukemia: A Case Report

**DOI:** 10.7759/cureus.40180

**Published:** 2023-06-09

**Authors:** Ekaterina Proskuriakova, Anuradha Sakhuja, Sarah Khan, Dhan B Shrestha, Pam Khosla

**Affiliations:** 1 Internal Medicine Department, Mount Sinai Hospital, Chicago, USA; 2 Hematology and Oncology Department, Mount Sinai Hospital, Chicago, USA; 3 Hematology and Oncology Department, Mount Sinai Hosipital, Chicago, USA

**Keywords:** braf v600e mutations, hairy cell leukemia, cladribine, spontaneous splenic rupture, unusual presentation of hairy cell leukemia

## Abstract

Hairy cell leukemia (HCL) represents a rare B-cell malignancy with 2% of all leukemias and should be differentiated from HCL-like conditions, including HCL-variant (HCL-V) and splenic diffuse red pulp lymphoma (SDRPL). HCL gets its name from the short, thin projections that look like hair on its cells. It is associated with a specific immunophenotypic profile, cytopenia, and splenomegaly. Spontaneous splenic rupture can be a symptom of hematological malignancy such as HCL and is a life-threatening acute emergency. Here, we present a case of a 37-year-old man who presented to the hospital with signs of acute peritonitis and acute anemia and was found to have atraumatic splenic rupture secondary to splenomegaly. He underwent emergent angiography, where the bleeding splenic vessel was identified, and the patient was successfully treated with embolization. Immunophenotypic profile revealed that B-cells were positive for CD11c, CD103, CD25, and CD5, for which he received five days of cladribine and achieved complete clinical remission.

## Introduction

Hairy cell leukemia (HCL) is a rare hematologic condition representing about 2% of all leukemias, with approximately 1,100 new cases reported annually in the United States [1]. The overall incidence of HCL is four times higher in males compared to females, with a median age of diagnosis of 55 years old. Although it may be seen at a younger age, it has rarely been diagnosed in children [2].

It gets its name from the short, thin projections that look like hair on its cells. Hairy cells tend to accumulate in the bone marrow, liver, and spleen. Even though HCL affects the white cells, the lymph nodes usually don’t enlarge [3]. HCL should be differentiated from other HCL-like conditions like HCL-variant (HCL-V) and splenic diffuse red pulp lymphoma (SDRPL). This differentiation could be done by morphological identification of hairy cells on the peripheral blood smear, searching for a specific immunophenotypic profile with clonal expansion of specific markers of B-cells (CD11c, CD103, CD123, and CD125) on peripheral blood or/and bone marrow and a different clinical course of the disease [3]. The immunologic score of 3 or 4 based on immunologic markers is observed in up to 98% of cases with HCL and 0-1 in other HCL-like disorders [4]. Another important factor separating HCL from other diseases is a well-known underlying mechanism of development, such as the mutation of B-Raf proto-oncogene (BRAF gene) that is identified in 70-100% of HCL cases [5]. BRAF somatic mutation is an essential molecular hallmark of HCL as it leads to new diagnostic and therapeutic options for patients with its presence [6].

Patients with HCL usually present with infections, splenomegaly, or cytopenia on complete blood count (CBC). However, manifestations such as autoimmune disease or unusual cases mimicking multiple myeloma also could be seen [7]. Asymptomatic patients can be monitored closely for the progression of the disease with CBC and physical evaluation every three to six months. Indications for treating patients with HCL include hemoglobin (Hgb) level, <11 g/dL; platelet count, <100/μL; and absolute neutrophil count (ANC), <1,000/μL or if they have symptomatic splenomegaly [3]. Current first-line treatment for HCL includes purine nucleoside analogs (PNA), cladribine, and pentostatin. Another choice will be PNA with subsequent rituximab therapy [8]. Due to its less toxic profile, cladribine is usually preferred as the first option in most cases, with complete response (CR) to therapy in up to 90% of patients after one cycle (five to seven days). Splenectomy could be considered an option for those with symptomatic splenomegaly, pancytopenia secondary to splenic sequestration, or pregnant women as a temporal measure [9].

We present a case of a 37-year-old male admitted to the hospital with acute abdominal pain following an atraumatic splenic rupture in a setting of HCL.

## Case presentation

A 37-year-old healthy man with no significant past medical history presented to the emergency department with acute abdominal pain localized to the left upper quadrant that awakened him from sleep. The patient also reported that he had been experiencing progressive abdominal discomfort for three months before this critical event. He does not smoke, drinks socially, and has no significant family history of malignancy. On the initial assessment, his abdomen was tender. Labs were notable for platelets of 60,000/μL, severe anemia with Hgb of 6.6 g/dL, and normal white blood count (WBC) of 7,700/μL (Table 1).

**Table 1:** Laboratory findings of the patient. g/dL, grams per deciliter; μL, microliter

Laboratory findings	On admission	Range
White blood cells (WBC)	7,700/μL	4,000-11,000/μL​​​​​​​
Red blood cells (RBC)	2,47 x 10^6^/μL​​​​​​​​​​​​​​	4.34-5.6 x 10^6^/μL​​​​​​​​​​​​​​​​​​​​​
Hemoglobin (Hbg)	6.6 g/dL	13.5-17.5 g/dL
Hematocrit (Hct)	23.5%	38.6-49.2%
Smudge cells	1+	
Platelets	60,000/μL​​​​​​​	150,000-450,000/μL​​​​​​​

**Figure 1:**
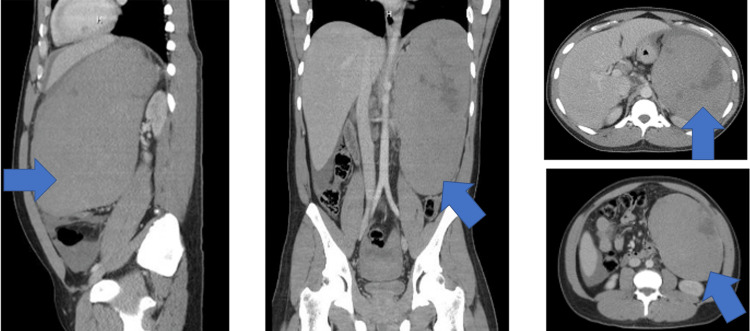
Computed tomography: lateral, coronal, and cross-sectional views of the abdomen with findings concerning massive splenomegaly measuring approximately 25 cm (blue arrow). Irregular hypoattenuating areas within the inferolateral and superolateral aspects of the spleen with small to moderate volume hemoperitoneum.

No fever, chills, dysuria, or gastrointestinal complaints were reported. Computed tomography (CT) of the abdomen displayed massive splenomegaly measuring approximately 25 cm with a hemoperitoneum with a high suspicion of atraumatic, spontaneous splenic rupture (Figure 1).

The patient was admitted to the hospital and initially underwent emergent embolization of the splenic artery. Meanwhile, his peripheral blood smear was sent for flow cytometry. It revealed the presence of clonal CD20+ B-cells with strong expression of CD11c, CD25, and CD103; partial CD5; and surface lambda light chain comprising 93% lymphocytes and approximately 89% of all analyzed white blood cells. The suspected diagnosis of HCL was confirmed.

The patient was given pneumococcal, meningococcal, and influenza vaccinations and was discharged from the hospital with further management with a five-day course of cladribine. A bone marrow biopsy was scheduled, and BRAF mutation testing was ordered. 

Before starting cladribine, the patient was given allopurinol for the prevention of tumor lysis syndrome (TLS). On day three of treatment, he developed severe neutropenia with ANC of 0.1 cells/µL, for which he was given Neulasta, Levaquin, Bactrim prophylaxis, and Acyclovir, with the improvement of blood cell count in two weeks. 

The spleen was shrinking slowly. Two and four months after the embolization, the CT scan displayed a 22 cm and an 18 cm spleen, respectively. A heterozygous mutation in BRAF gene V600E was detected, which provided an option to consider vemurafenib and Rituxan for this patient if there were a partial response to the treatment with cladribine. However, after 11 months since the initial presentation and course of cladribine, the spleen was not palpable, and the bone marrow biopsy did not show any clonal evidence of leukemia. Currently, the patient is in complete clinical remission.

## Discussion

HCL is a rare, slow-growing leukemia that starts in a B-cell (B lymphocyte). Genetic errors lead to uncontrolled growth and division of the B-cells. HCL presentation can be different and will include fatigue in most cases (80%), infection (30%), bleeding or bruising (30%), abdominal pain due to splenomegaly (25%), lymphadenopathy (17%), and B symptoms such as weight loss or night sweats. However, patients may sometimes present asymptomatic [10]. The median duration of symptoms ranges from four to six months [11], and the median age of patients with HCL is considered to be between 47 and 62 years [12]. Our patient presented with abdominal pain due to splenomegaly that has been worsening for four months, which is consistent with the standard features of HCL, but the age of our patient was slightly below the median age among patients with this disease.

In a rare 2% of cases, HCL may present as focal, lytic bone lesions (FBL). This finding usually occurs in the late phase of the disease and is associated with the more severe and aggressive stage. Bone lesions are usually treated with standard therapy or can be managed sometimes with site radiotherapy [13]. Extranodal involvement can occur in up to 15% of cases with many involved locations. Some of the rare sites of extranodal involvement are considered central nervous system (CNS), typically treated with either cladribine or rituximab as they can cross the blood-brain barrier [14].

HCL can also present with a rare complication like a spontaneous splenic rupture [15]. A spontaneous rupture is a common event in an enlarged spleen, like in our patient with a massive splenomegaly measuring approximately 25 cm. It is characterized as a spleen rupture without any preceding trauma. The rupture mechanism could be explained by organ congestion caused by the accumulation of blasts and hemorrhage due to coagulation pathology and infarction of the spleen.

The initial laboratory evaluation of HCL includes a CBC that can display cytopenia. The most commonly found cytopenia is neutropenia in up to 80% of patients, while thrombocytopenia will be seen in 70% and anemia in 30% of patients with HCL. On the other hand, lymphocytosis can rarely be seen in just 10% of patients [10]. In our case, the patient presented with low thrombocytes and Hgb on his CBC, while his WBC was normal. Imaging is not frequently used in HCL, and response to treatment is usually assessed with CBC/bone marrow assessment. As our patient presented with acute abdominal pain, we used CT to assess his condition and later evaluate his spleen size after spleen artery embolization (SAE). SAE is a minimally invasive procedure with a short recovery period and does not need general anesthesia, allowing salvage of the spleen, which is one of the essential organs of the immune system [16]. Therefore, after SAE, immunization should be undertaken to prevent sepsis, meningitis, or pneumonia triggered mainly by Streptococcus pneumoniae, Neisseria meningitidis, and Haemophilus influenzae type b [17].

The definitive diagnosis of HCL is usually associated with the expression of CD19, CD20, CD22, CD200, CD11c, CD103, CD123, and CD25 markers. About 98% of people with HCL will express around three to four of the last four markers, while our patient had CD11c, CD25, CD103, and CD5. In addition, our patient had a BRAF V600E mutation that could be identified in 70-100% of cases with HCL [3]. Chemotherapy with PNA is the primary treatment for patients with HCL with the first line-therapy of either cladribine or pentostatin. In phase II clinical trial that included 59 untreated patients with HCL, a CR of 100% was achieved. Moreover, adding rituximab to cladribine showed improvement in the duration of CR [18]. Our patient achieved CR with a single-agent purine analog and currently is in complete remission.

## Conclusions

Most patients with HCL will present with fatigue, infections, or bleeding. Spontaneous splenic rupture can be one of the rare presentations of HCL. It is a life-threatening acute emergency preceded by splenomegaly associated with the left upper quadrant pain and fullness. It can be treated with emergent embolization.

HCL is a rare, slow-growing leukemia that starts in a B-cell and has a unique sensitivity to purine analogs, cladribine, and pentostatin, the treatment of choice. Patients usually have high response rates and durable remission.
